# A study on the grey relation analysis between the overall performance and individual event scores of international elite trampolinists

**DOI:** 10.3389/fspor.2024.1373481

**Published:** 2024-09-10

**Authors:** Xinyu Zhu, Lejun Wang

**Affiliations:** ^1^ Sport College, Jiujiang University, Jiujiang, Jiangxi, China; ^2^ Sport and Health Research Center, Shanghai YangZhi Rehabilitation Hospital (Shanghai Sunshine Rehabilitation Center), Physical Education Department, Tongji University, Shanghai, China

**Keywords:** trampoline, competition results, international elite trampoline athlete, grey relational analysis (GRA), 2020 Tokyo Olympics

## Abstract

This study applied grey relational analysis to assess the relationship between individual trampoline event scores and overall performance of top male and female athletes in the 2020 Tokyo Olympics. Analyzing execution, horizontal, difficulty, timing of flight, and total scores, results showed males excelled significantly in difficulty, timing, and overall performance, while execution and horizontal scores were comparable. For males, timing of flight (excluding outliers) had the greatest influence on total score, followed by difficulty and execution. In females, difficulty dominated, followed by timing and horizontal, with execution least impactful. The study highlighted the primary roles of timing and difficulty scores in overall trampoline performance, with gender variations in score contributions. The findings illuminated the interplay of score components, offering a theoretical framework for targeted trampoline training. For international athletes, key considerations included boosting height index for a robust trampoline foundation; tailoring difficulty levels to athletes' abilities while adhering to scoring rules, without sacrificing technical prowess; and sustaining training to refine quality and stability of routines.

## 1 Introduction

Trampoline, often referred to as the “aerial ballet”, is a highly challenging and aesthetically pleasing sport that combines competition, artistic expression, spectator engagement, and entertainment. The outcomes of trampoline competitions are determined by various sub-scores, including the execution score, difficulty score, and timing of flight score. These individual sub-scores not only contribute to the overall results but also serve as significant indicators of athletes' exceptional abilities across different dimensions (1, 2). With the introduction of the “horizontal new rule” since the 2017–2020 Olympic cycle, a new horizontal score of 10 points (H score) was added on the basis of the previous three scoring criteria: the execution score, the difficulty score, and timing of flight scores. This addition helps to evaluate athletes' overall performance in competitions more comprehensively, encompassing their in-air displacement control, stability, and rebound effect after landing, which are used to assess the precision of their landing points. Concurrently, the execution score was reduced from the previous 30 points to 20 points. The introduction of the horizontal displacement score has refined and enhanced the accuracy of the scoring system. Judges can now provide more objective and fair scores by observing athletes' displacement during their bounces, thereby reducing the influence of subjective judgments on the scoring results.

These rule changes have altered the determination of competition results, making the relationship between sub-scores and the total score more intricate. However, there is currently a lack of research and discussion on the relationship between sub-scores and the total score of international trampoline athletes after the introduction of the “horizontal new rule”.

Against this backdrop, the present study aims to analyze the performance of athletes in trampoline competitions during the Tokyo 2020 Olympic Games, following the implementation of the “Horizontal New Rule”. The focus is to uncover the distinctive characteristics and interdependencies within the performance composition of elite international trampoline athletes under the influence of the “Horizontal New Rule”. The advent of the new displacement regulations has ushered in a more objective scoring system for trampoline competitions, mitigating the impact of subjective judgments by referees. This evolution not only fosters an environment where athletes can fully leverage their unique strengths but also enriches the diversity of the sport, ushering in a new era of fairness and inclusivity in trampoline contests. This analysis involves examining the relationship between the execution score, difficulty score, timing of flight score, horizontal score, and total score. The insights gained from this study can serve as valuable references for the scientific training of trampoline athletes.

## 2 Methods

### 2.1 Study design

The primary focus of this study is to analyze the performance of trampoline athletes during the Tokyo 2020 Olympic Games, specifically after the implementation of the “Horizontal New Rule”. To ensure the reliability of the competition results and the inclusion of a substantial number of athletes, the research and analysis utilize the results of male and female trampoline athletes who demonstrated exceptional competence in the finals, without significant errors. The criterion for judging “significant errors” for each athlete is that the athlete can't finish the whole 10 skills due to errors in competitions, which has been determined by observing the public competition videos. The judging has been conducted by a specific operator of a trampoline referee. The game scores used in this study are sourced from the officially published scorebook (3), thereby ensuring the accuracy and validity of the analyzed performance data. Grey Relation Analysis has been conducted to determine the relationship between each sub-score (execution score, horizontal score, difficulty score, timing of flight score) and the overall score in trampoline competitions. Moreover, statistical analysis was used to compare the differences in scores between male and female groups.

### 2.2 Observation based on competition videos

We conducted observation and analysis based on the publicly available standard competition videos. Subsequent to the competition, detailed observation and analysis of the recorded videos facilitated the identification of the technical movement characteristics exhibited by the athletes. In order to ensure the accuracy of the analysis, performance data from athletes who made significant errors were excluded from the dataset.

### 2.3 Grey relation analysis

Grey relational analysis is utilized to calculate the degree of grey relation between each sub-score (execution score, horizontal score, difficulty score, timing of flight score) and the overall score in trampoline competitions. The correlation degrees are subsequently ranked based on the influence of each sub-score on the total score. The calculation of the grey relation degree follows established methodologies as outlined in relevant literature (3–5).

The basic steps are as follows:

(1) Establish the mother-child sequence, where the trampoline competition results serve as the mother sequence (Y) and the trampoline execution score(X1), horizontal score (X2), difficulty score (X3), and time of flight score (X4) from the sub-sequence.
(2) Standardize the original data by dividing each sequence by its respective average.
(3) Calculate the absolute difference between each sub-sequence and the mother sequence, and record the corresponding values.
(4) Determine the correlation coefficient by substituting the corresponding differences (ΔX' = Y − Xn), the minimum difference (Δmin), and the maximum difference (Δmax) into the formula for calculating the grey relation coefficient.

i.e.,

(1) $$\mathrm{corr}\;=\;\frac{\Delta \min\;+\;\rho \Delta \max}{\Delta X^{\prime }\;+\;\rho \Delta \max}$$

According to previous research, we used *p* = 0.5 in this study (4).

Finally, grey correlation degree was determined by the following equation:

(2) $$r\;=\;\frac{1}{m}\sum_{n\;=\;1}^{m}n\;\;\times \;\mathrm{corr}\;(n)$$

### 2.4 Statistical analysis

The Kolmogorov-Smirnov test was employed to evaluate the normality of data distribution, which indicated that the data did not follow a normal distribution. Consequently, a non-parametric test for two independent samples was utilized to compare the differences in scores between male and female groups. Statistical analyses were performed using SPSS for Windows version 13.0 (SPSS Inc., Chicago, IL, USA). All significance thresholds were set at *α* = 0.05.

## 3 Results

**Figure 1:**
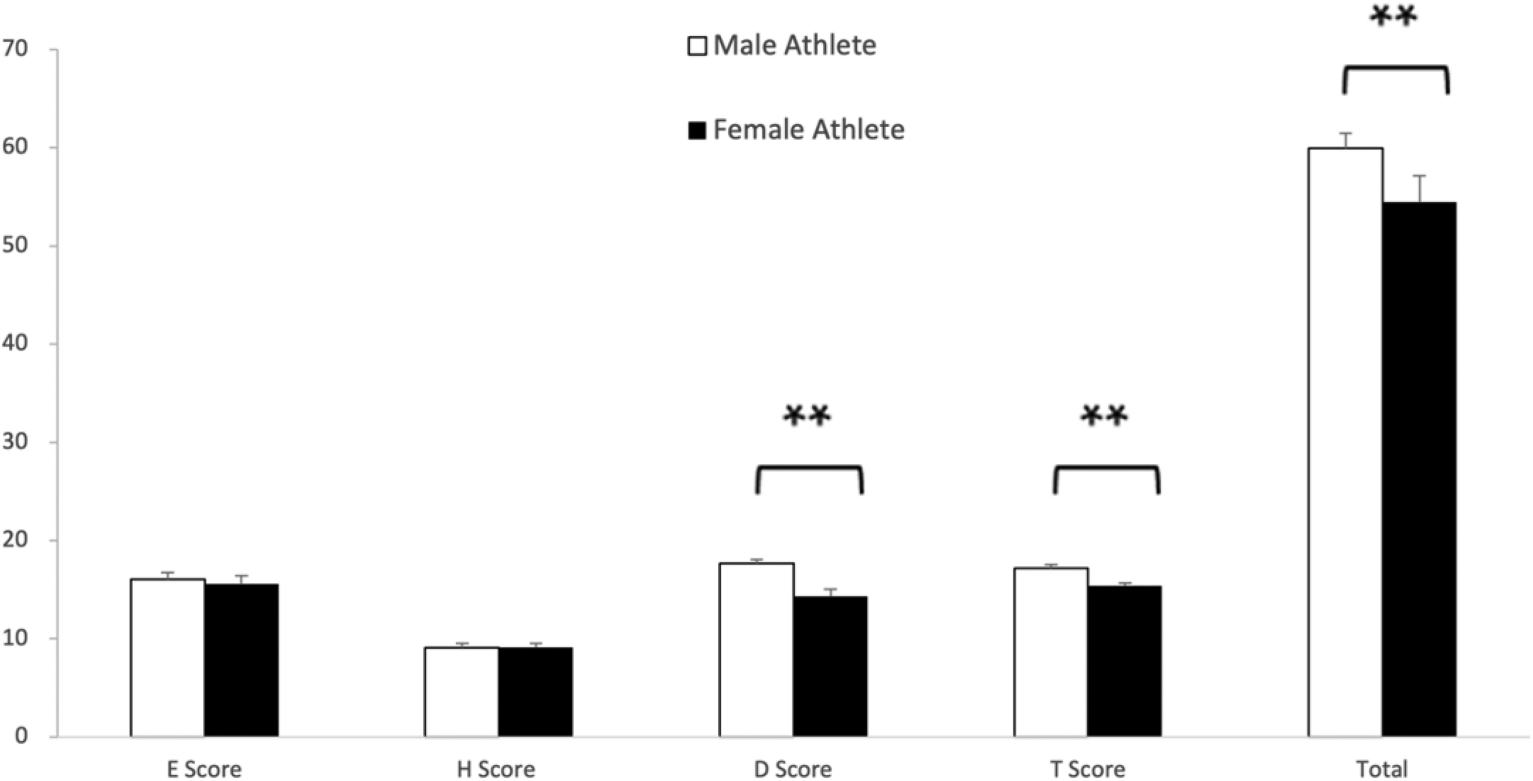
Comparison of each sub-item score and total score between male and female group trampoline athletes. (D score, T score and total score were significantly higher in male group athletes than female group. **indicates *P* < 0.01.).

Tables 1, 2 presented the scores of the primary athletes (without major mistakes) in the men's and women's trampoline finals at the 2020 Tokyo Olympic Games, respectively. The average scores and standard deviations of each result for each group of athletes were calculated, as depicted in Figure 1.

**Table 1:** Main athletes’ scores in the men's trampoline final of Tokyo 2020 Olympic games.

No.	E score	H score	D score	T score	Total
1	16.500	9.100	18.200	17.915	61.715
2	16.600	9.700	17.800	17.135	61.235
3	16.300	9.600	17.800	16.975	60.675
4	16.300	9.000	17.900	17.365	60.565
5	16.500	8.900	17.100	17.100	59.600
6	15.200	8.500	17.800	16.735	58.235
7	14.900	8.700	17.300	16.915	57.815

**Table 2:** Main athletes’ scores in women's trampoline final of Tokyo 2020 Olympic games.

No.	E score	H score	D score	T score	Total
1	16.800	9.400	15.000	15.435	56.636
2	16.000	9.100	15.000	16.250	56.350
3	15.800	9.400	15.000	15.535	55.735
4	15.600	9.200	14.900	15.760	55.460
5	16.000	9.300	14.000	15.355	54.655
6	15.300	9.300	14.300	15.450	54.350
7	15.300	9.000	14.400	15.590	54.290
8	13.900	8.000	12.800	13.645	48.345

As observed in Figure 1, the execution score, horizontal score, difficulty score, timing of flight score, and total score of male trampoline athletes were 16.04 ± 0.69, 9.07 ± 0.44, 17.70 ± 0.37, 17.16 ± 0.39, and 59.98 ± 1.49, respectively. For women, the scores were 15.59 ± 0.83, 9.09 ± 0.46, 14.43 ± 0.76, 15.38 ± 0.75, and 54.48 ± 2.63, respectively.

Significant differences were observed between male and female trampoline athletes in terms of difficulty scores, timing of flight scores, and total scores. Male athletes achieve significantly higher scores in these three categories compared to female athletes. However, there was no significant difference in the execution scores and horizontal scores between the genders.

Table 3 provides the grey relation degree and ranking list of the scores and total scores for male and female trampoline athletes. It can be seen that the grey correlation degree between each sub-item score and the total score of male and female trampoline athletes were all above 0.5, suggesting a significant relationship between each sub-item and the total score (4). For male athletes, among the for sub-item score, the timing of flight score had the highest grey relation with the total score, following the difficulty score, the execution score, and the horizontal score. For female athletes, among the for sub-item score, the difficulty score had the highest grey relation with the total score, following the timing of flight score, execution score and the horizontal score.

**Table 3:** The grey relation degree and ranking list of each sub-item score and total score of male and female trampoline athletes.

	Categories	E score	H score	D score	T score
Grey corr	Male	0.668	0.530	0.706	0.708
	Female	0.578	0.621	0.739	0.652
Rank	Male	3	4	2	1
	Female	4	3	1	2

## 4 Discussion

Grey relation analysis is a method used to assess the proximity of analyzed data series to a standard series curve. This analysis quantifies the influence or contribution of the analyzed data series to the standard data series (6). It provides a straightforward and intuitive approach for evaluating and quantitatively analyzing the effects of multiple factors (7). Grey relation analysis is widely utilized in various fields, including the diagnosis of athletes' competitive ability (8–10), evaluation of physical fitness (11–13), and studying the relationship between scores of different items and total scores (7–15).

By employing grey relation analysis in this study, we can assess the degree of influence that each sub-score has on the total score in trampoline competitions. This analysis helps to elucidate the relative importance of different factors and their contributions to the overall performance of trampoline athletes.

Trampoline is classified as a technical sport characterized by intricate performances. The modern competitive trampoline is defined by its emphasis on being “high, difficult, accurate, stable, and beautiful”, which are crucial factors for achieving success in the sport (15). With the implementation of new scoring rules in trampoline competitions, the main scoring items have evolved from the previous focus on height, difficulty, and technique to include the horizontal score as the fourth component. These four sub-items in trampoline competitions provide a comprehensive assessment of athletes' technical movements, highlighting the importance of achieving height, demonstrating difficulty, maintaining accuracy, and exhibiting stability. These qualities are fundamental requirements for trampoline athletes in order to excel in their performances.

“Height” in trampoline refers to the consistency of achieving vertical height in individual maneuvers as well as the overall height of the routine (16). Adequate vertical height not only increases the score for the height component of the routine but also allows sufficient airtime for completing the routine itself (16). Thus, height plays a dual role in trampoline competitions and has become a crucial factor in determining success. A study conducted by Li Jian (1) revealed that following the implementation of timing of flight scoring rules in 2011, the development of individual men's and women's trampoline events exhibited varying characteristics, but ultimately led to comprehensive improvements in difficulty score, execution score, and total score. In another study by Peng Yuanzhi et al. (4), a grey relation analysis was conducted on the performance of eight male trampoline athletes in the finals of the 28th World Trampoline Championships in 2011. The analysis demonstrated that the correlation between the timing of flight score and the overall performance score was the strongest, indicating a highly significant connection between the timing of flight score and the total score in trampoline sports. In the present study, the grey relation analysis revealed that the timing of flight score had the highest ranking in terms of its relationship with the total score for male trampoline athletes, while for female trampoline athletes, it held the second position. This finding indicates a close relationship and significant contribution of the timing of flight score to the overall score of both male and female trampoline athletes.

“Difficulty” in trampoline refers to the complexity and difficulty level of the maneuvers performed, as well as the assigned difficulty value of the routine (16). In modern competitive trampoline sports, the competition rules encourage athletes to incorporate more challenging and intricate maneuvers in order to enhance the spectacle of the sport and promote its development. Notably, the grey relation values of the difficulty score and the total score rank among the top two components, suggesting a significant overall impact and contribution of the difficulty score to the performance score of both male and female trampoline athletes in international competitions. This finding differs from previous studies (17) and can be attributed to the international emphasis placed on the difficulty of trampoline maneuvers, aligning with the prevailing direction of the competition rules. Theoretically, an increase in the difficulty score will inevitably affect the height, execution quality, and stability of the maneuvers, consequently influencing the timing of flight score, execution score, and horizontal score (18). Therefore, the increase in difficulty score for technical maneuvers represents a double-edged sword for trampoline athletes, necessitating careful consideration of its impact on other scoring components and the overall score during training and competition. Achieving better results in international trampoline competitions requires a delicate balance between the technical execution of maneuvers and the assigned difficulty value of the routine. Athletes must carefully navigate the interplay between these factors to optimize their overall performance and score.

“Accuracy” in trampoline primarily refers to the precision and exactness displayed in technical maneuvers, including takeoff, mid-air flips, descent, landing on the mat, and contact with the net (16). The execution score, which evaluates the accuracy and quality of the athlete's maneuvers, specifically measures the level of “accuracy” achieved in comparison to the standard maneuvers. Previous research has highlighted the significance of difficulty as the foundation, height as the guarantee, and the quality of execution as the key factors for success in trampoline competitions (19). The grey relation analysis conducted on difficulty score, execution score, timing of flight score, and total score in trampoline sports reveals noteworthy findings. For male trampoline athletes, the grey relation between the execution score and the total trampoline score attains a value of 0.668, ranking third in terms of its impact on the total score. In contrast, for female trampoline athletes, the grey relation between the execution score and the total trampoline score reaches 0.578, ranking fourth in terms of its impact on the total score. This indicates that among international elite trampoline athletes who have successfully avoided significant errors, the contribution of the execution score to their overall score is relatively lower. This observation could be attributed to the high level of accuracy and quality achieved by these athletes in executing their routine maneuvers, resulting in a comparatively reduced impact on the final total score.

“Stability” in trampoline refers to the consistency of a trampoline athlete's technical performance, which is primarily evaluated based on factors such as the degree of horizontal displacement at the landing point and the occurrence of errors or failures during the routine execution (16). The introduction of the horizontal score as a scoring component in 2017 aimed to quantify the stability of an athlete's landing position. In this study, as it focuses on athletes without significant errors during the competition, the horizontal score effectively reflects the “stability” of their technical routines. The grey relation analysis reveals that the grey relation coefficients between the horizontal score and the total score for male and female trampoline athletes are 0.530 and 0.621, respectively. These values rank fourth and third in terms of their impact on the total score. This indicates that the horizontal score does have an influence on the total score, but its significance is relatively lower among international elite trampoline athletes. In trampoline competitions, the horizontal score of athletes is susceptible to fluctuations due to environmental and psychological factors, which can affect the quality of routine execution and overall performance (20–22). Therefore, the impact of the horizontal score on the total score is not solely reflected in the score itself but also significantly influences the quality of routine execution and even the successful completion of the routine. This highlights the crucial role played by the horizontal score in determining the total score (23). However, for high-level international trampoline athletes, since the introduction of the “Horizontal New Rule” in 2017, they have placed emphasis on training the accuracy of the landing point, which was previously overlooked in regular training. For technically proficient trampoline athletes, their exceptional spatial positioning skills and sense of orientation are sufficient to easily achieve accuracy in landing, provided that the focus is placed on the precision of the landing point alongside the accuracy of routine execution.

Based on the analysis conducted, it is evident that the height, difficulty, execution, and horizontal scores respectively capture the characteristic features of trampoline sports, namely “height, difficulty, accuracy, and stability”, which are crucial for achieving success in competitions. Among these individual scores, height and difficulty have a significant impact on the total score, while execution and horizontal scores have a relatively minor influence. Furthermore, the rankings of these scores reveal gender differences between male and female trampoline athletes in terms of the total score. Following the implementation of the new trampoline rule incorporating the horizontal displacement score (H score), the phenomenon of male athletes significantly outperforming female athletes in terms of difficulty scores, timing of flight scores, and total scores can be primarily attributed to male athletes’ advantages in selecting more challenging routines, physical fitness, technical proficiency, and competition strategies. Male athletes tend to opt for more difficult maneuvers as a means of self-challenge, whereas female athletes may select slightly less difficult routines due to considerations of technical stability, physical limitations, or strategic planning. Furthermore, male athletes generally possess physical advantages, including strength, power, and jumping ability, which facilitate their attainment of higher difficulty and height scores. Nevertheless, in terms of execution score and H score, the differences between male and female athletes are not pronounced, reflecting athletes' emphasis on adapting to the new rules and maintaining technical consistency. In the future, as athletes continue to adapt to the new rules and enhance their skills, these disparities may undergo changes.

Based on these research findings, several considerations should be taken into account for trampoline athletes, particularly those preparing for international events:

1. Emphasize the development of the height index to establish a solid foundation for executing trampoline routines.
2. While focusing on improving the height index, customize the difficulty values of individual and combined routines based on the latest scoring rules and individual differences of the athletes, without compromising technical stability.
3. Maintain training to enhance the quality and stability of routines for these exceptional trampoline athletes.

However, it is important to note that the grey relation index only quantifies the degree of proximity between the curves representing individual scores and the total score, measuring the impact and contribution of individual scores to the total score. In this study, all the grey relation coefficients between individual scores and the total score were above 0.5, indicating a significant influence and contribution of the variations in individual scores to the total score. Nevertheless, due to the complex interactions and interconnectedness of various factors contributing to victory in sports, dominant factors in one aspect of athletes' skill development cannot wholly replace other factors. Instead, they should be optimized and integrated to form a cohesive and efficient whole, enabling athletes to reach their optimal competitive state (15).

Therefore, in sports training, while emphasizing the development of specific individual scores, it is essential to consider the holistic impact of these changes on other individual scores and the total score. This approach ensures that the training system remains systemic and maintains the dynamic balance of athletes' skill levels. By doing so, a more scientifically and logically organized training plan can be established.

## 5 Conclusion

With the implementation of the “Horizontal New Rule”, male trampoline athletes exhibited significantly higher difficulty scores, timing of flight scores, and total scores compared to their female counterparts. However, there were no notable differences in completion scores and horizontal scores between the genders. The results of the grey relation analysis further highlighted that timing of flight scores and difficulty scores had a greater impact on the total score, while completion scores and horizontal scores had a relatively lesser influence. Moreover, the analysis revealed gender disparities in the contributions of these sub-scores to the total score.

These findings underscored the importance of different sub-scores in evaluating overall performance and shed light on their interplay. They provided valuable theoretical insights and presented avenues for targeted training and improvement of trampoline athletes, enabling them to achieve exceptional results in international competitions.

Given the significant positive impact of height and difficulty scores on the overall performance, athletes and coaches should prioritize training in these two areas. For male athletes, it is crucial to continue enhancing the difficulty and height of their movements, exploring even more challenging technical maneuvers while also emphasizing psychological resilience development and tactical strategy training. For female athletes, on the other hand, they can design training programs that focus on enhancing strength, explosiveness, and technical complexity while maintaining technical stability, gradually attempting to increase movement difficulty and jump height. With advancements in technology, leveraging high-tech equipment and data analysis software to conduct real-time monitoring and precise analysis of athletes' training and competition processes can facilitate a deeper understanding of the underlying mechanisms of trampoline athletes' skill development, thereby providing a more scientific and comprehensive theoretical foundation for training.

## Data Availability

The original contributions presented in the study are included in the article/Supplementary Material, further inquiries can be directed to the corresponding author.
